# Atypical Presentation and Neuroradiological Features of Giant Ecchordosis Physalyphora in a Seven-Year-Old Patient: A Case Report

**DOI:** 10.7759/cureus.23544

**Published:** 2022-03-27

**Authors:** Anas Raffa

**Affiliations:** 1 Radiology, King Abdulaziz University Faculty of Medicine, Jeddah, SAU

**Keywords:** proton beam therapy, mri, giant ecchordosis physaliphora, ct, brain tumor

## Abstract

This study presented the rare case of a seven-year-old patient with atypical presentation and neuroradiological features of giant ecchordosis physaliphora. The patient underwent cross-sectional imaging due to persistent headache without neurological or visual symptoms. CT scan imaging of the head revealed a hypodense tumor in the prepontine cistern. This lesion caused smooth scalloping of the dorsal clivus without aggressive erosion or calcification, and an osseous stalk was also identified between the lesion and the dorsal wall of the clivus. Sagittal T1 weighted image (T1WI) MRI showed a bilobed, solid and cystic, well-defined lesion, measuring 3.5 cm in terms of craniocaudal diameter, found alongside the midline within the prepontine cistern. After the evaluation of radiological images, the patient was then subjected to endoscopic transnasal complete tumor excision. Histological examination revealed sheets and lobules of clear cells with cytoplasmic globules "physaliphorous cells", and myxoid stroma. There was nuclear pleomorphism associated with focal areas of necrosis. After full recovery and discharge, the patient was followed up for the first year with four-month interval brain MRI scans showing no evidence of residual tumors. On the 12 months follow-up scan, the MRI scan revealed a 1.5 x 0.7 cm recurrent mass in the retroclival right paramidline prepontine cistern, which was most notably seen on the diffusion-weighted images. Series of proton beam therapy with annual MRI scans demonstrated regression of the tumor, eventually allowing the patient to live free of neurological symptoms up to this day. Results suggest that the utilization of radiological imaging such as CT and MRI scans was successful in identifying the ecchordosis physaliphora and differentiating it from chordomas. It can also be inferred that atypical radiological and histopathological findings of ecchordosis physaliphora lesions might suggest that they are prone to recurrence, which is an atypical feature for such entities. Further studies are recommended to explore and better understand these uncommon observations in patients with ecchordosis physaliphora.

## Introduction

In 1856, German pathologist Hubert von Luschka first described the presence of pathologic ectopic notochordal tissue at the dorsal clivus [1]. Controversy exists regarding the nomenclature of this entity in the literature in which both intradural chordomas and ecchordosis physaliphora are referred to as the same pathology. The term "intradural chordoma" was proposed by Wolfe et al. [2] for all notochordal remnant tumors. Rodriguez et al. [3] suggested that "ecchordosis physaliphora" should be used to refer to all intradural lesions found at notochordal remnant origin that have not been pathologically proven to be chordomas. In the present, these two entities are recognized as different pathologies but belong to a common origin [3].

Differentiation between ecchordosis physaliphora and its pathologic counterpart chordoma can be achieved based on radiological findings. Ecchordosis physaliphora includes fluid attenuation on CT scan and lack of contrast enhancement or aggressive osseous erosion. Chordomas, on the other hand, typically present as an extra-axial soft tissue hyperdense lesion having osseous erosion, compression on the brain stem (described as a thumb sign), heterogeneous T2 weighted image (T2WI) signal intensity, and varying degrees of contrast enhancement. Intrinsic tumor calcification might be present [2,3].

This case report presented the atypical presentation and neuroradiological features of a giant ecchordosis physaliphora in a seven-year-old patient.

## Case presentation

**Figure 1:**
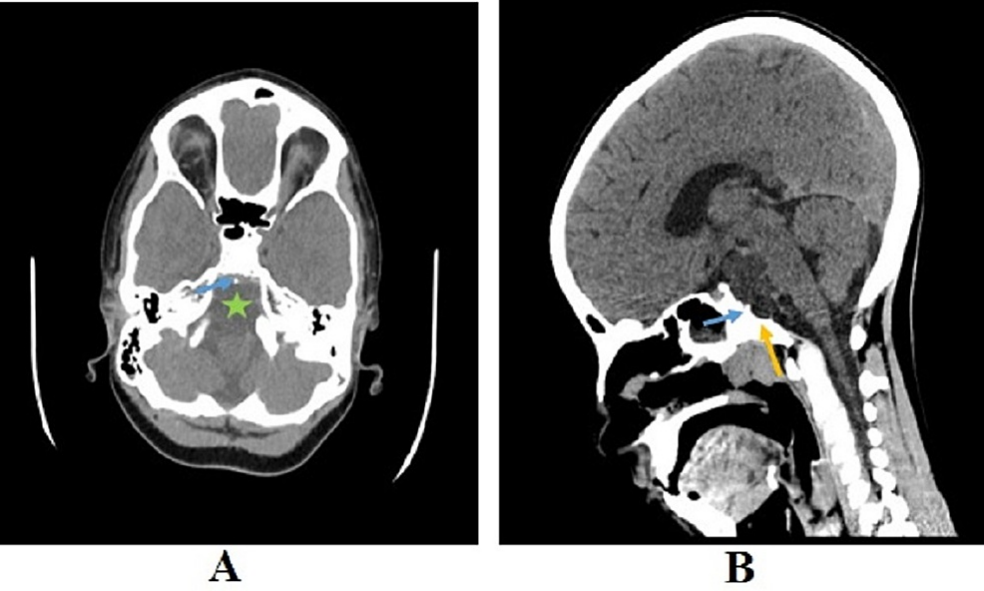
CT scans of the patient CT scans of the patient show (A) hypodense lesion in the prepontine cistern, without calcification (green star), osseous stalk (blue arrow; axial soft tissue window CT image), and (B) smooth scalloping of the posterior clivus (orange arrow) and osseous stalk (blue arrow; sagittal unenhanced CT image).

A seven-year-old male patient was subjected to cross-sectional imaging due to persistent headache without neurological or visual symptoms on April 21st, 2016. The patient was medically free until the onset of symptoms and did not have a family history of brain tumors. CT scan imaging revealed a hypodense brain tumor in the prepontine cistern causing smooth scalloping of the dorsal clivus without aggressive erosion or calcification (Figures 1A). An osseous stalk was also identified between the lesion and dorsal wall of the clivus (Figure 1B).

**Figure 2:**
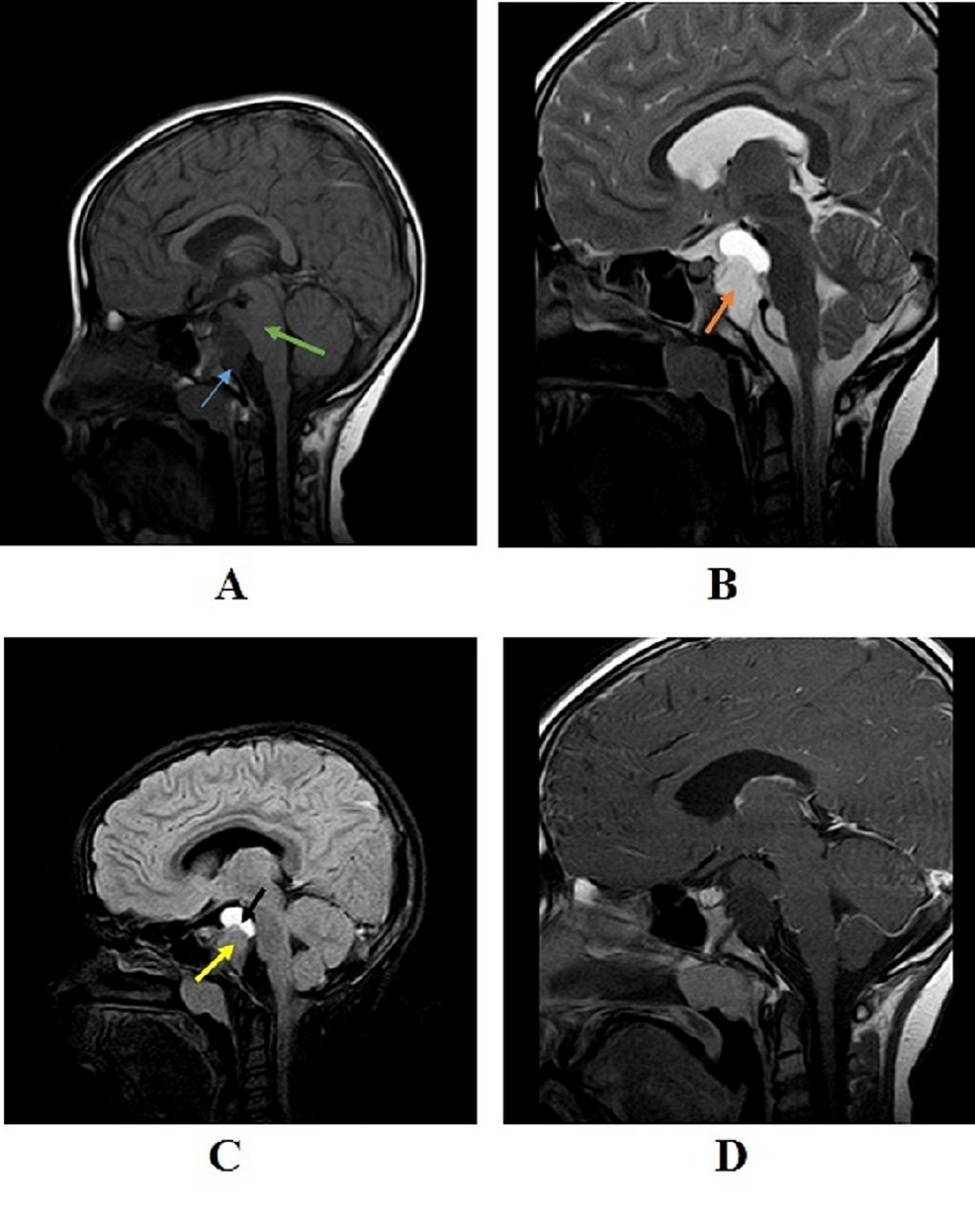
MRI scans of the patient MRI scans of the patient with (A) sagittal T1WI showing bilobed solid and cystic intradural lesion in the prepontine cistern (blue arrow), and cystic component showing isointense signal (green arrow); (B) sagittal T2WI showing hyperintense signal of the solid and cystic components (orange arrow); (C) sagittal fluid-attenuated inversion recovery (FLAIR) showing isointense signal of the solid (yellow arrow) and hyperintense signal of the cystic component (black arrow); and (D) sagittal T1 post-gadolinium showing no enhancement in the lesion.

Brain MRI sagittal T1 weighted image (T1WI) revealed a bilobed, solid and cystic, well-defined lesion, measuring 3.5 cm in terms of craniocaudal diameter, found alongside the midline within the prepontine cistern. This lesion mildly compresses the right side of the pons and displaces the basilar artery. Also, the lesion had an oblong shape and was centered over the posterior border of the clivus intradurally. Analysis of the radiological images revealed the superior part of the cystic component on T1WI to exhibit isointense signal (Figure 2A). The isointense signal of the solid area was also observed on fluid-attenuated inversion recovery (FLAIR) on its sagittal view (Figure 2C), while its cystic component exhibited a hyperintense signal. Hyperintense signal was also observed on sagittal T2WI (Figure 2B). The post-gadolinium (post-GAD) images did not show any contrast enhancement (Figure 2D).

**Figure 3:**
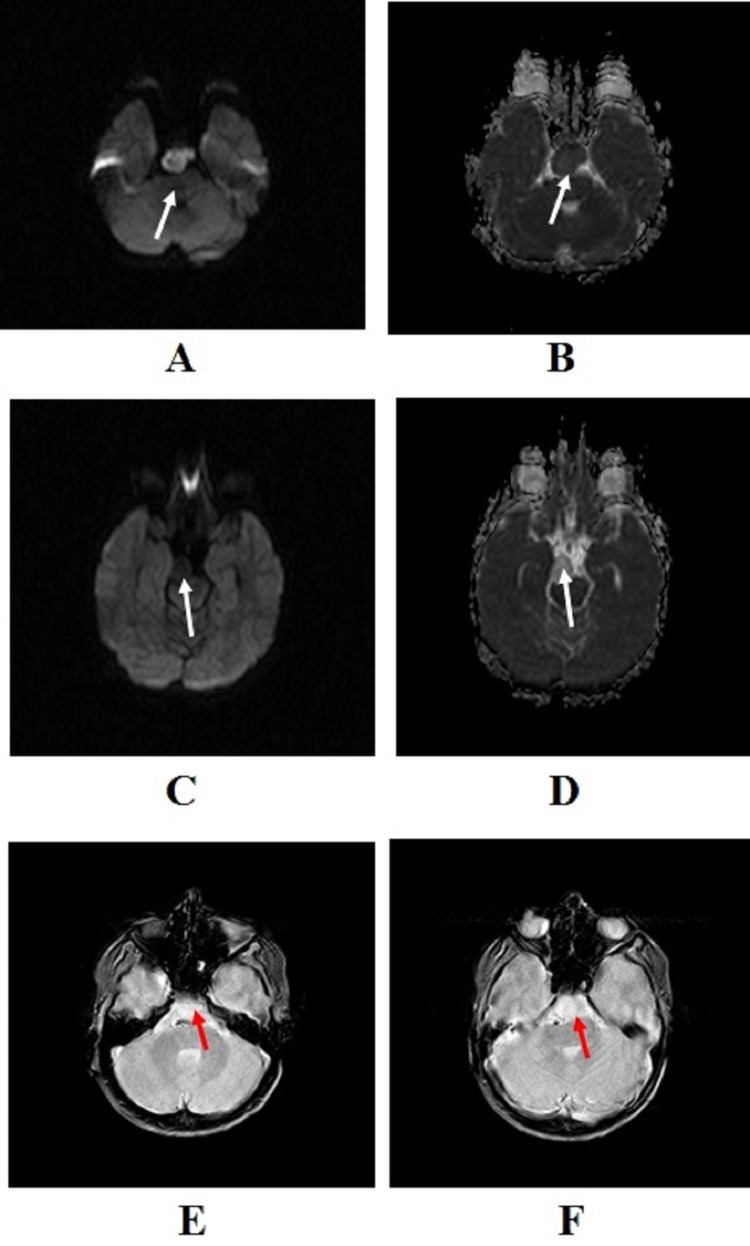
Scans of the patient Illustration of (A) diffusion-weighted imaging (DWI) and apparent diffusion coefficient (ADC) scans showing restricted diffusion (white arrow); (B) DWI and ADC scans showing no restriction on the cystic portion (white arrow); (C) gradient-echo sequence showing no susceptibility within the solid or cystic part of the lesion (white arrow); and (D-F) smaller cystic component on T1WI  showing no restricted diffusion, and susceptibility artifacts.

The illustration of diffusion-weighted imaging (DWI) and apparent diffusion coefficient (ADC) imaging scans revealed restricted diffusion within the solid part of the lesion (Figures 3A-B). However, DWI and ADC imaging scans show no restriction on the cystic portion. Gradient echo sequence shows no susceptibility within the solid or cystic part of the lesion (Figure 3C). In addition, the smaller cystic component on its superior part on T1WI showed no restricted diffusion, and susceptibility artifact (Figures 3E-F)

After the evaluation of radiological images, the patient was then subjected to endoscopic transnasal complete tumor excision. Histological examination revealed sheets and lobules of clear cells with cytoplasmic globules "physaliphorous cells", and myxoid stroma. There was nuclear pleomorphism associated with focal areas of necrosis. Immunohistochemistry staining was positive for AE1/AE3, epithelial membrane antigen (EMA), vimentin, and focally for S100. Ki-67 shows <3% positive staining of the cells in focal areas. Cytokeratin 7 (CK7), cytokeratin 20 (CK20), and CDX-2 staining were negative. The final histopathology report provided the differential diagnosis of notochordal origin tumor and thereby advised correlation with the clinical and radiological results.

**Figure 4:**
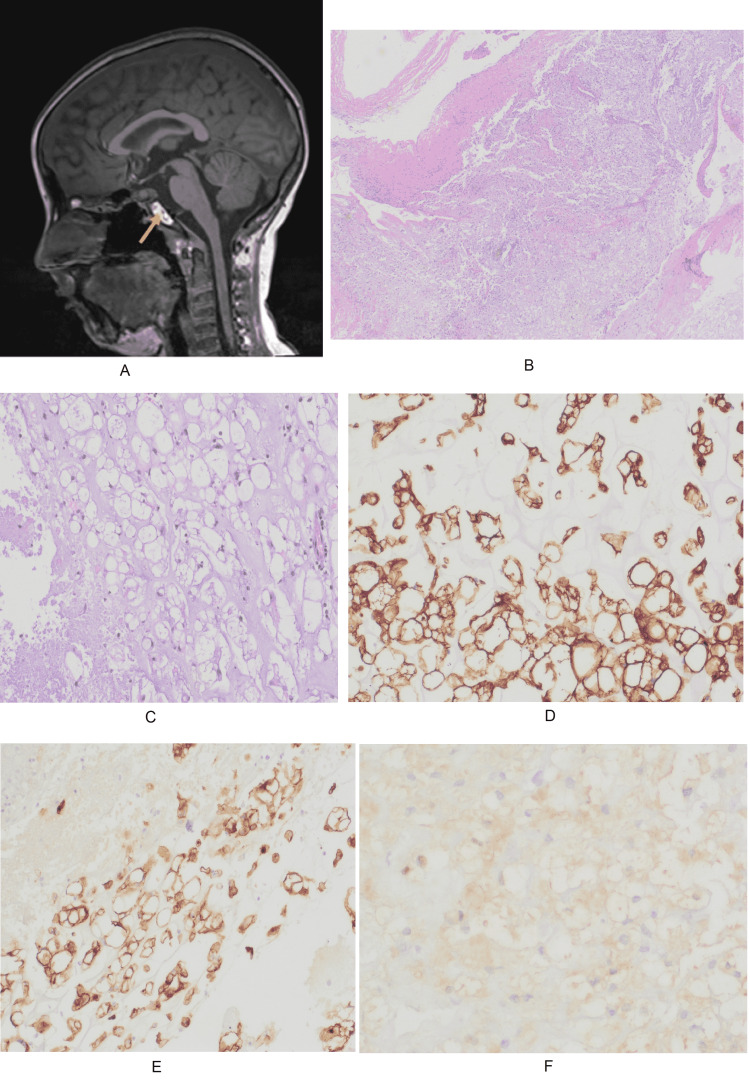
Postoperative MRI scan and results of histopathological examination (A) Postoperative four-month follow-up MRI scan (sagittal unenhanced T1 weighted image) of the patient showing postsurgical blood (yellow arrow) after complete resection of the tumor without residual tissue. Histopathological examination shows sheets and lobules of clear cells with myxoid stroma (B) and cytoplasmic globules "physaliphorous cells" and nuclear pleomorphism (C). Histological staining was positive for AE1/AE3 (D), epithelial membrane antigen (E), and S100 (F).

The postoperative course was uneventful. After recovery and discharge, the patient was regularly followed up for the first year by clinic visits, and four-month interval brain MRI scans, where he remained free from headache or neurological symptoms, and the MRI scan did not show evidence of residual or recurrent tumors (Figure 4).

**Figure 5:**
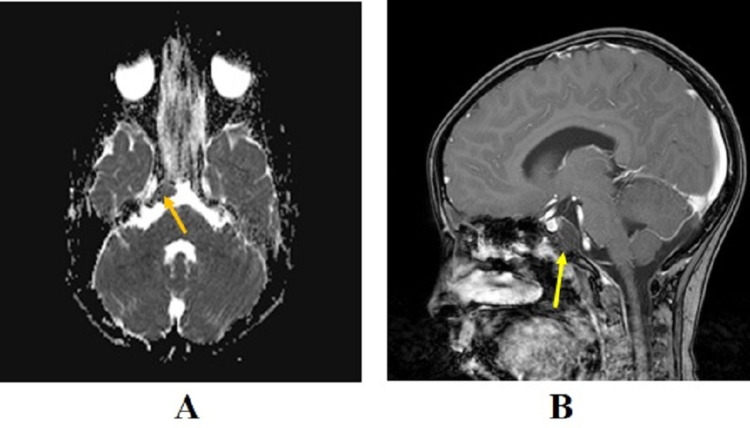
Postoperative 12-month follow-up MRI scans of the patient Postoperative 12-month follow-up MRI scans of the patient with (A) diffusion-weighted image (DWI) showing recurrent lesion with restricted diffusion on the retroclival prepontine cistern (orange arrow); and (B) sagittal post-gadolinium T1 weighted image (T1WI) showing no tumor enhancement (yellow arrow).

However, on the 12-month follow-up scan, the MRI scan revealed a 1.5 x 0.7 cm recurrent mass in the retroclival right paramidline prepontine cistern, which was most notably seen on the diffusion-weighted images (Figure 5A) and sagittal post-GAD T1WI (Figure 5B) sequences. Given the location of the recurrent tumor, surgical resection was not possible.

Consequently, the primary physician and radiation oncology team, along with the family's approval, decided to begin treatment utilizing a sum of 73.8 Gy (RBE) 41 fractions (1.8 Gy [RBE]) of proton beam irradiation, with planar kV images to ascertain positioning and regular interval contrast MRI during treatment. The clinical target volume was at the skull base region of the recurrent tumor, and a 2 mm planning target volume was included at the margin. Proton therapy was well tolerated without any signs of treatment-related side effects. The first follow-up MRI was obtained three months after completion of therapy and did not show a significant change in the tumor. This was also followed up by a six-month MRI, and later a series of annual MRI scans, auditory, ophthalmological and hormonal tests. Radiological imaging revealed favorable results by the regression of the tumor size. Six years after the initial presentation, the patient is headache-free without neurological symptoms and doing well to this day.

## Discussion

The tumor, ecchordosis physaliphora (EP), is an uncommon congenital benign hamartomatous-type which is detected for about 2% of post-mortem examinations [4,5]. Described as tiny, gelatinous-like tissue, it is considered to be an ectopic notochordal remnant [2,3,6,7]. The location for these tumors is commonly the retroclival prepontine cistern space in the middle cranial fossa, attaching to the dorsal wall of the clivus through a tiny pedicle [2,7,8]. Other locations involve the midline starting from the base portion of the skull down to the sacrum [4,8]. These lesions are typically formed in notochordal remnants [5]. Commonly, these lesions are asymptomatic and detected incidentally [8] due to their slow growth and tiny size. However, EP can be symptomatic and may occasionally produce symptoms and signs from compressive effects on the brainstem or cranial nerves [4,5,9].

In terms of classification by size, lesions larger than 6 cm^3^ in volume or 3 cm in diameter are already referred to as giant EP according to the literature [5]. There are only four reported cases of giant EP in the dorsal clival area of the prepontine cistern [10-13], having the reported lesion sizes to range between 20 mm and 40 mm. Of these four cases, three patients were male, and only one was female, having an average age of 28 years old (youngest was 12 years old, oldest was 63 years old). Diplopia and headache were reported to be the major presenting symptoms.

With regards to diagnosis, EP cannot be differentiated based on histopathological evaluation. Its confirmation is done based on the imaging characteristics [8]; however, it imposes limitations. Cerebrospinal fluid signal (CSF) density of the lesion and presence of posterior fossa streak artefacts limit the sensitivity of CT scan for detecting such lesions. A well-demarcated nonaggressive cortical defect of the dorsal clivus wall can be seen on a CT scan. The presence of an osseous stalk at the base of the lesion connecting it to the clivus is considered pathognomonic for this entity [5,8]. These lesions follow CSF signal on T1WI, T2WI, and fluid-attenuated inversion recovery (FLAIR) sequences and do not demonstrate contrast enhancement, unlike chordomas [5,8]. Describing the radiological imaging characteristics, scans revealed that the lesions exhibited a hypointense signal on T1WI as well as a hyperintense signal on T2WI without contrast enhancement for the previously reported four studies of giant EP [10-13]. As observed in our study, a small bone stalk developing from the retroclivus was also described in the case report of Krisht et al. [11]. In addition, it is also important to assess these lesions on contrast-enhanced images, as none of the recorded EP cases show significant enhancement [3,8,9,14-16].

In differentiating between EP and chordomas, the observation that they are indistinguishable based on histology and immunohistochemistry supports that both have a common notochordal origin [2,7,17,18]. Although histological sparse pleomorphism, hypocellularity, and absence of mitoses [9,19] could help differentiate the two entities, these are still not considered criteria for the diagnosis. Currently, the proliferation rate marker, MIB-1 labeling index, is considered to be useful in correlating recurrences in chordomas as well as distinguishing it from EP [9,14,18,20]. However, it remains unclear whether an EP can transform into a chordoma [3,17,18].

## Conclusions

This study presented a rare case of a giant ecchordosis physaliphora in a young patient with a unique characteristic of cystic degeneration. The utilization of radiological imaging such as CT and MRI scans was successful in identifying the ecchordosis physaliphora and differentiating it from chordomas. Series of proton beam therapy with annual MRI scans favored the regression of the tumor size, eventually allowing the patient to live free from neurological symptoms up to this day. In addition, results suggest that atypical ecchordosis physaliphora lesions might be prone to recurrence, which is an unusual feature for such entities. Further studies are recommended to explore and better understand these uncommon observations in patients with ecchordosis physaliphora.
